# Awareness, willingness to use, and history of HIV PrEP use among gay, bisexual, and other men who have sex with men in Nigeria

**DOI:** 10.1371/journal.pone.0226384

**Published:** 2019-12-18

**Authors:** Adedotun Ogunbajo, Stella Iwuagwu, Rashidi Williams, Katie Biello, Matthew J. Mimiaga

**Affiliations:** 1 Department of Behavioral and Social Sciences, Brown University School of Public Health, Providence, Rhode Island, United States of America; 2 Center for Health Equity, Brown School of Public Health, Providence, Rhode Island, United States of America; 3 Centre for Right to Health, Abuja, Nigeria; 4 Equality Triangle for Health and Peoples Development Initiative, Warri, Delta, Nigeria; 5 Department of Epidemiology, Brown University School of Public Health, Providence, Rhode Island, United States of America; 6 The Fenway Institute, Fenway Health, Boston, Massachusetts, United States of America; 7 Department of Psychiatry and Human Behavior, Brown University Alpert Medical School, Providence, Rhode Island, United States of America; Northwestern University, UNITED STATES

## Abstract

**Background:**

Nigerian gay, bisexual, and other men who have sex with men (GBMSM) are disproportionately affected by HIV, with an estimated prevalence of between 11–35%. Pre-exposure prophylaxis (PrEP) has the potential to significantly decrease incident HIV infections among Nigerian GBMSM. Understanding the relationship between socio-demographic, sexual risk behavior, and psychosocial factors with PrEP awareness, willingness to use, and history of use among this group is pivotal to maximizing PrEP uptake.

**Methods:**

Between March and June 2019, 419 participants completed an interviewer-administered survey assessing PrEP awareness, willingness to use, and history of use; socio-demographics; sexual risk behavior; and psychosocial factors. Bivariate and multivariable logistic regression were used to examine factors associated with PrEP awareness, willingness to use, and history of use.

**Results:**

53.6% were aware of PrEP; 80.1% were willing to use PrEP; and 29.7% had previously used PrEP. In multivariable analysis, factors associated with an increased odds of PrEP awareness include residing in Abuja [adjusted odds ratio (aOR) 5.02; 95% confidence interval (CI): 2.13 to 11.83] and Lagos (aOR 12.30; 95% CI: 4.92 to 30.67) vs. residing in Plateau, living with HIV (aOR 2.56; 95% CI: 1.54 to 4.72), using location-based apps for seeking sexual partners (aOR 4.06; 95% CI: 2.28 to 7.24), having health insurance (aOR 2.31; 95% CI: 1.08 to 4.40), history of suicidal thoughts (aOR 2.05; 95% CI: 1.02 to 4.10), and history of PrEP use (aOR 45.5; 95% CI: 5.60 to 370.04). Decreasing clinically significant depressive symptoms was associated with lower willingness to use PrEP (aOR 0.96; 95% CI: 0.92 to 0.99). Lastly, factors associated with increased odds of having a history of PrEP use were those reporting 6 or more insertive anal sex acts in the last 30 days compared to those with none (aOR 5.76; 95% CI: 1.58 to 20.98) and being aware of PrEP (aOR 29.6; 95% CI: 3.78 to 231.84).

**Discussion:**

Nearly half of the Nigerian GBMSM in this study had no prior awareness of PrEP, but after being informed about its potential benefits, the majority were willing to use it. However, PrEP uptake among Nigerian GBMSM remains low. Findings suggest that educational messages are necessary to ensure appropriate PrEP scale-up, especially tailored towards Nigerian GBMSM.

## Introduction

Nigeria has the fourth largest human immunodeficiency virus (HIV) epidemic in the world, with a prevalence of 1.4% among the general adult population and an estimated 1.9 million people living with HIV [1]. Nigerian gay, bisexual and other men who have sex with men (GBMSM) bear a disproportionately higher burden of HIV, with an estimated prevalence of 11–35% [2]. Oral pre-exposure prophylaxis (PrEP) is a biomedical prevention approach that significantly reduces HIV acquisition when taken daily [3–5]. Given its’ efficacy, PrEP has the potential to significantly decrease new HIV infections among Nigeria GBMSM. In 2013, a consultation of community leaders, implementers, and academics from17 African countries gathered to assess HIV prevention priorities for GBMSM and identified oral PrEP as a major biomedical prevention strategy [6]. Currently, PrEP is not widely available in Nigeria, however there are two ongoing implementation and demonstration projects with an estimated 400–600 individuals currently taking PrEP [7].

Gaining an in-depth understanding of awareness of and acceptability of PrEP among Nigerian GBMSM is crucial for preparing for PrEP uptake, once it becomes more widely available. To date, only a few studies have investigated PrEP awareness, acceptability, and current use among African GBMSM. A recently published study of 459 HIV-negative Kenyan GBMSM found that 64.3% had awareness of PrEP and 44.9% were willing to use PrEP in the future [8]. Another study of 137 GBMSM in Ghana found low awareness but high acceptability of PrEP [9]. These studies provide preliminary evidence of PrEP being acceptable by African GBMSM.

To date, there are no known published studies that have explored the relationship between socio-demographic, sexual risk behavior, and psychosocial factors with PrEP awareness, willingness to use PrEP, and history of PrEP use for HIV prevention among Nigerian GBMSM. The goal of the current study is to examine these relationships among a community-recruited sample of GBMSM in four diverse regions of Nigeria. Understanding these relationships will be helpful for designing successful intervention programs to increase PrEP uptake among this marginalized group.

## Methods

### Participants and procedures

Between March and June 2019, 413 GBMSM enrolled in the study and completed a quantitative assessment. Inclusion criteria were: 1) 18 years of age or older; 2) currently residing in one of four Nigerian states (Abuja, Lagos, Delta or Plateau); 3) cis-gender male; and 4) having sex with another male.

Participants were recruited through community-based organizations (CBOs) in each of the four states and via snowball sampling. Outreach workers and peer educators at the respective CBOs shared information about the study with the target population during programming events (e.g., HIV testing and counseling, health education, advocacy events, etc.) and provided study contact information to individuals who were interested. Eligible participants were then asked to provide study information to their peer networks.

Study activities took place in private offices within our partner CBOs. The study was approved by the institutional review boards at Brown University and the Nigerian Institute of Medical Research. Informed verbal consent was obtained from each participant prior to enrolling them in study. Each participant completed an interviewer administered behavioral survey. Participants were compensated 4,000 Naira (10 US dollars) for their participation.

### Measures

#### Demographics

Participants were asked their age, relationship status, education level and sexual orientation.

#### Social marginalization

Participants were asked their monthly income, employment status, financial hardship, and any history of incarceration.

#### Sexual health

Participants were asked their sexual position, HIV status, and any history of sexually transmitted infections (STIs), any STIs in the last year, number of receptive and insertive anal sex partners in the last 30 days, condom use at last anal sex, and any usage of geosocial networking apps.

#### Healthcare access

Participants were asked whether or not they had a current primary care provider (yes/no), health insurance (yes/no), and whether they had been unable to access healthcare in the last year due to costs (yes/no).

#### Mental health

Participants were asked about depressive symptoms using the Center for Epidemiologic Studies Depression Scale (CES-D) [10], a 20-item scale used to screen for clinically significant depressive symptoms. The items were scored on a 4-point scale from 0–3, with a higher score indicating higher levels of depressive symptoms. Previous suicide thoughts were assessed by asking participants: “Have you ever thought about ending your life or committing suicide?” (yes/no). Internalized homophobia was assessed using a three-item scale scored on 5-point Likert scale [11], with a higher score indicating higher levels of internalized homophobia.

#### Substance use

Participants were asked about their alcohol and drug use. Problematic alcohol use was assessed with the AUDIT-C [12], a 3-item screening for heavy drinking or alcohol dependence. The AUDIT-C is scored on a scale of 0–12; a score of 4 or greater indicated hazardous drinking. Participants were asked about having ever used recreational drugs, including (marijuana, tramadol, rophynol (flunitrazepam), codeine, poppers (alkyl nitrites), and cocaine) (yes/no).

#### PrEP awareness, willingness to use PrEP and history of using PrEP

To assess PrEP awareness, participants were asked: “Have you heard of pre-exposure prophylaxis (PrEP) for HIV prevention?” (yes/no). To assess willingness to use PrEP, participants were provided some brief information about PrEP (i.e., PrEP is one pill taken once daily by HIV-negative people to reduce their risk of HIV infection) and then asked: “Would you be willing to take an oral pill every day to prevent getting HIV infection?” (willing/not willing/not sure, dichotomized into willing/not willing or not sure). We excluded participants who self-reported currently living with HIV as there are not eligible to take PrEP. To assess history of PrEP use, participants were asked: “Have you ever taken PrEP to prevent HIV?” (yes/no). We excluded participants who self-reported living with HIV and those currently residing in Delta or Plateau state, where PrEP is currently unavailable.

### Data analysis

We assessed the distribution (percentages and means) of all variables by PrEP awareness, willingness to use, and history of use. Chi-square global tests of independence were used to assess independent association between variables. Bivariate logistic regression was conducted to assess the association between socio-demographic, sexual risk behavior, and psychosocial factors and the three PrEP-related outcomes (PrEP awareness, willingness to use PrEP, and history of PrEP use). It was determined that a sample size of 400 GBMSM (100 recruited in each of the four states) would provide 80% power to detect a 30% or greater difference in PrEP awareness between the four States sampled at a two sided .05 significance level. Variables significant at the p<0.05 level in the bivariate models were retained in each of the three multivariable models assessing these outcomes. Data were analyzed using SAS version 9.4 (Cary, NC).

## Results

As seen in Table 1, Participants ranged in age from 18 to 60 years (mean = 29.2, SD = 5.8), with 59.4% identifying as bisexual, and 62.3% were single. More than a third (37.7%) of the sample was living with HIV and 32.1% had an STI diagnosis in the last year. Almost half (48.8%) had a primary care provider but only 15.6% had health insurance. The depression scores ranged from 0 to 60 with a mean score of 10.0 (SD = 11.5); 23.7% of participants had a score of 16 or higher, indicating moderate to severe depression. 18.9% of participants were indicated for hazardous drinking and history of drug use were prevalent: marijuana (42.2%), tramadol (15.2%), rophynol (15.1%), codeine (24.1%), poppers (8.6%), cocaine (7.1%). More than half the sample (53.6%) were aware of PrEP, 80.1% were willing to use PrEP, and 29.7% had a history of PrEP use.

**Table 1:** Socio-demographic, sexual risk behavior, and psychosocial factors associated with PrEP awareness, willingness to use PrEP, and history of PrEP use among Nigerian GBMSM from March-June 2019 (N = 413).

	Total Sample (N = 413)	Awareness of PrEP (n = 401)		P	Willingness to Use PrEP (n = 251)		P	PrEP Use (n = 138)		P
		Aware of PrEP (n = 215, 53.6%)	Unaware of PrEP (n = 186, 46.4%)		Willing (n = 201, 80.1%)	Not Willing/Not Sure (n = 50, 19.9%)		History of PrEP Use (n = 41, 29.7%)	No history of PrEP Use (n = 97, 70.3%)	
**Site**										
Abuja	104 (26.1)	74 (71.2)	30 (28.8)	**<0.0001**	39 (73.6))	14 (26.4)	0.49	18 (33.3)	36 (66.7)	0.54
Delta	100 (25.1)	31 (31.0)	69 (69.0)		56 (84.9)	10 (15.1)		-	-	
Lagos	109 (27.4)	91 (83.5)	18 (16.5)		64 (79.0)	17 (21.0)		23 (28.4)	58 (71.6)	
Plateau	85 (21.4)	19 (22.4)	66 (77.7)		39 (81.3)	9 (18.7)		-	-	
***Socio-Demographics***										
**Age**										
Mean (SD)	29.2 (5.80)	29.9 (5.16)	29,7 (7.09)	0.72	28.9 (5.80)	30.8 (5.74)	**0.04**	28.0 (5.60)	29.6 (5.74)	0.13
**Relationship Status**										
Single	250 (62.3)	146 (58.4)	104 (41.6)	**0.01**	130 (83.3)	26 (16.7)	0.09	30 (32.9)	61 (67.0)	0.24
Not Single	151 (37.7)	69 (45.7)	82 (54.3)		71 (74.7)	24 (25.3)		11 (23.4)	36 (76.6)	
**Education**										
SSS or lower	182 (45.4)	78 (42.9)	104 (57.1)	**<0.0001**	86 (81.1)	20 (18.9)	0.46	16 (27.1)	43 (72.9)	0.67
Some Uni or Vocation Sch	86 (21.5)	40 (46.5)	46 (53.5		46 (83.6)	9 (16.4)		6 (27.3)	16 (72.7)	
College degree or higher	109 (27.2)	83 (76.2)	26 (23.9)		57 (79.2)	15 (20.8)		14 (30.4)	32 (69.6)	
Other	24 (6.0)	14 (56.3)	10 (41.7)		12 (66.7)	6 (33.3)		5 (45.5)	6 (54.5)	
**Sexual Orientation**										
Gay/Homosexual	162 (40.6)	92 (56.8)	70 (43.2)	0.26	68 (76.4)	21 (23.6)	0.30	17 (31.5)	37 (68.5)	0.56
Bisexual	237 (59.4)	121 (51.1)	116 (48.9)		131 (81.9)	29 (18.1)		22 (26.8)	60 (73.2)	
**Monthly Income (in Naira)**										
0–10000	103 (26.0)	50 (48.5)	53 (515)	0.008	47 (85.5)	8 (14.5)	0.20	7 (21.9)	25 (78.1)	0.35
10,000–30,000	106 (26.7)	46 (43.4)	60 (56.6)		62 (84.9)	11 (15.1)		12 (35.3)	22 (64.7)	
30,000–50,000	79 (19.9)	44 (55.7)	35 (44.3)		41 (75.9)	13 (24.1)		11 (40.7)	16 (59.3)	
50,000–100,000	56 (14.1)	35 (62.5)	21 (37.5)		24 (80.0)	6 (20.0)		5 (29.4)	12 (70.6)	
100,000+	52 (13.1)	37 (71.2)	15 (28.8)		23(67.7)	11 (32.3)		5 (19.2)	21 (80.8)	
**Employment Status**										
Employed	323 (80.5)	169 (52.3)	154 (47.7)	0.29	159 (79.5)	41 (20.5)	0.65	31 (30.4)	71 (69.6)	0.77
Unemployed	78 (19.5)	46 (59.0)	32 (41.0)		42 (82.4)	9 (17.6)		10 (27.8)	26 (72.2)	
**Financial Hardship**										
Yes	241 (60.7)	128 (53.1)	113 (46.9)	0.79	119 (82.1)	26 (17.9)	0.39	26 (33.3)	52 (66.7)	0.22
No	156 (39.3)	85 (54.5)	71 (45.5)		80 (77.7)	23 (22.3)		14 (23.7)	45 (76.3)	
**History of Incarceration**										
Yes	88 (22.2)	46 (52.3)	42 (47.7)	0.77	34 (87.2)	5 (12.8)	0.21	2 (11.8)	15 (88.2)	0.09
No	309 (77.8)	167 (54.0)	142 (46.0)		164 (78.5)	45 (21.5)		38 (31.9)	81 (68.1)	
***Sexual Health***										
**Sexual Position**										
Bottom/Versatile Bottom	108 (27.4)	60 (55.6)	48 (44.4)	0.82	50 (83.3)	10 (16.7)	0.61	9 (24.3)	28 (75.7)	0.37
Versatile	118 (29.9)	64 (54.2)	54 (45.8)		57 (81.4)	13 (18.6)		11 (40.7)	16 (59.3)	
Versatile Top/Top	168 (42.6)	87 (51.8)	81 (48.2)		89 (77.4)	26 (22.6)		21 (30.0)	49 (70.0)	
**Self-Reported HIV Status**										
Positive/Unknown	151 (37.7)	91 (60.3)	60 (39.7)	**0.04**	N/A	N/A	N/A	N/A	N/A	N/A
Negative	250 (62.3)	124 (49.6)	126 (50.4)		N/A	N/A	N/A	N/A	N/A	N/A
**Any History of STIs**										
Yes	176 (44.0)	103 (58.5)	73 (41.5)	0.090	81 (80.2)	20 (19.8)	0.95	16 (37.21)	27 (62.8)	0.21
No	224 (56.0)	112 (50.0)	112 (50.0)		119 (79.9)	30 (20.1)		25 (26.6)	69 (73.4)	
**STIs in last year**										
Yes	127 (32.1)	70 (55.1)	57 (44.9)	0.62	56 (78.9)	15 (21.1)	0.81	13 (41.9)	18 (58.1)	0.08
No	269 (67.9)	141 (52.4)	128 (47.6)		142 (80.2)	35 (19.8)		27 (25.7)	78 (74.3)	
**# of Receptive Anal sex acts in the last 30 days**										
0	179 (44.8)	87 (48.6)	92 (51.4)	**0.05**	96 (78.7)	26 (21.3)	0.95	16 (23.5)	52 (76.5)	0.31
1	56 (14.0)	33 (58.9)	23 (41.1)		28 (80.0)	7 (20.0)		6 (37.5)	10 (62.5)	
2–3	82 (20.5)	42 (51.2)	40 (48.8)		46 (83.6)	9 (16.4)		12 (41.4)	17 (58.6)	
4–5	52 (13.0)	28 (53.9)	24 (46.1)		21 (77.8)	6 (22.2)		3 (18.8)	13 (81.2)	
6+	31 (7.8)	24 (77.4)	7 (22.6)		9 (81.8)	2 (18.2)		3 (37.5)	5 (62.5)	
**# of Insertive Anal sex acts in the last 30 days**										
0	134 (33.7)	68 (50.8)	66 (49.2)	0.69	66 (77.7)	19 (22.3)	0.87	8 (19.1)	34 (80.9)	**0.05**
1	50 (12.6)	26 (52.0)	24 (48.0)		26 (81.3)	6 (18.7))		4 (20.0)	16 (80.0)	
2–3	97 (24.4)	49 (50.5)	48 (49.5)		52 (83.9)	10 (16.1)		11 (30.6)	25 (69.4)	
4–5	55 (13.8)	33 (60.0)	22 (40.0)		29 (80.6)	7 (19.4)		6 (33.3)	12 (66.7)	
6+	62 (15.6)	36 (58.1)	26 (41.9)		25 (75.8)	8 (24.2)		11 (55.0)	9 (45.0)	
**Condom Use last anal sex**										
Yes	310 (78.3)	181 (58.4)	129 (41.6)	**0.0005**	159 (78.7)	43 (21.3)	0.35	37 (30.8)	83 (69.2)	0.46
No	86 (21.7)	32 (37.2)	54 (62.8)		39 (84.8)	7 (15.2)		4 (22.2)	14 (77.8)	
**Geosocial App Usage**										
Yes	214 (53.4)	148 (69.2)	66 (30.8)	**<0.0001**	99 (80.5)	24 (19.5)	0.87	22 (29.3)	53 (70.7)	0.92
No	187 (46.6)	67 (35.8)	120 (64.2)		102 (79.7)	26 (20.3)		19 (30.2)	44 (89.8)	
***Healthcare Access***										
**Primary care Provider**										
Yes	196 (48.8)	112 (57.1)	84 (42.9)	0.17	96 (81.4)	22 (18.6)	0.63	21 (31.3)	46 (68.7)	0.68
No	205 (51.1)	103 (50.2)	102 (49.8)		105 (78.9)	28 (21.1)		20 (28.2)	51 (71.8)	
**Health Insurance**										
Yes	62 (15.6)	43 (69.3)	19 (30.7)	0.007	32 (74.4) 167 (81.1)	11 (25.6)	0.32	8 (29.6)	19 (70.4)	0.98
No	335 (84.4)	170 (50.8)	165 (49.2)			39 (18.9)		32 (29.4)	77 (70.6)	
**Unable to assess medical care due to cost in last year**										
Yes	173 (43.1)	83 (48.0)	90 (52.0)	**0.05**	81 (77.9)	23 (22.1)	0.57	11 (23.9)	35 (76.1)	0.29
No	228 (56.9)	132 (57.9)	96 (42.1)		120 (81.6)	27 (18.4)		30 (32.6)	62 (67.4)	
***Mental Health***										
**Depressive Symptoms (CESD-20)**	10.0 (11.5)	12.1 (13.0)	10.2 (11.0)	0.11	11.0 (12.0)	6.9 (7.9)	**0.02**	10.9 (11.5)	10.2 (11.6)	0.75
**Suicide Ideation**										
Yes	86 (21.5)	59 (68.6)	27 (31.4)	**0.002**	41 (85.4)	7 (14.6)	0.33	6 (22.2)	21 (77.8)	0.33
No	314 (78.5)	155 (49.4)	159 (50.6)		160 (79.2)	42 (20.8)		35 (31.8)	75 (68.2)	
**Internalized Homophobia**	7.1 (3.4)	7.26 (3.4)	8.66 (3.6)	**<0.0001**	7.84 (3.5)	7.88 (3.31)	0.94	7.3 (3.3)	7.3 (3.2)	0.99
***Substance Use***										
**Hazardous Drinking (AUDIT-C)**										
Yes	78 (18.9)	48 (63.2)	28 (36.8)	0.06	39 (73.6)	14 (26.4)	0.18	11 (31.4)	24 (68.6)	0.80
No	335 (81.1)	167 (51.4)	158 (48.6)		162 (81.8)	36 (18.2)		30 (29.1)	73 (70.9)	
**Lifetime Marijuana Use**										
Yes	168 (42.2)	98 (58.3)	70 (41.7)	0.10	83 (76.2)	26 (23.8)	0.15	20 (29.4)	48 (70.6)	0.89
No	230 (57.8)	115 (50.0)	115 (50.0)		116 (83.5)	23 (16.5)		19 (28.4)	48 (71.6)	
**Lifetime Tramadol Use**										
Yes	59 (15.2)	35 (59.3)	24 (40.7)	0.35	28 (80.0)	7 (20.0)	0.95	9 (45.0)	11 (55.0)	0.09
No	330 (84.8)	174 (52.7)	156 (47.3)		165 (80.5)	40 (19.5)		28 (26.2)	79 (73.8)	
**Lifetime Rohypnol Use**										
Yes	59 (15.1)	25 (42.4)	34 (57.6)	0.07	31 (81.6)	7 (18.4)	0.82	8 (38.1)	13 (61.9)	0.25
No	333 (84.9)	184 (55.3)	149 (44.7)		164 (80.0)	41 (20.0)		28 (25.7)	81 (74.3)	
**Lifetime Codeine Use**										
Yes	95 (24.1)	45 (47.4)	50 (52.6)	0.17	43 (76.8)	13 (23.2)	0.38	13 (38.2)	21 (61.8)	0.12
No	299 (75.9)	166 (55.5)	133 (44.5)		155 (82.0)	34 (18.0)		24 (24.2)	75 (75.8)	
**Lifetime Poppers Use**										
Yes	34 (8.6)	28 (82.4)	6 (17.6)	**0.0004**	14 (58.3)	10 (41.7)	**0.005**	3 (15.0)	17 (85.0)	0.15
No	369 (91.4)	183 (50.8)	177 (49.2)		184 (82.5)	39 (17.5)		35 (30.7)	79 (69.3)	
**Lifetime Cocaine Use**										
Yes	28 (7.1)	21 (75.0)	7 (25.0)	**0.02**	13 (61.9)	8 (38.1)	**0.03**	6 (37.5)	10 (62.5)	0.39
No	364 (92.9)	189 (51.9)	175 (48.1)		185 (81.9)	41 (18.1)		32 (27.1)	86 (72.9)	
***PrEP Indicators***										
**Awareness of PrEP**										
Aware of PrEP	215 (53.6)	N/A	N/A	N/A	94 (47.2)	105 (52.8)	0.17	39 (40.6)	57 (59.4)	**<0.0001**
Unaware of PrEP	186 (46.4)	N/A	N/A		29 (58.0)	21 (42.0)		1 (2.5)	39 (97.5)	
**Willingness to use PrEP**										
Willing	201 (80.1)	94 (47.2)	105 (52.8)	0.17	N/A	N/A	N/A	34 (32.1)	72 (67.9)	0.17
Not Willing/Not Sure	50 (19.9)	29 (58.0)	21 (42.0)		N/A	N/A		6 (19.4)	25 (80.6)	
**PrEP Use**										
History of PrEP Use	41 (29.7)	39 (97.5)	1 (2.5)	**<0.0001**	34 (85.0)	6 (15.0)	0.17	N/A	N/A	N/A
No History of PrEP Use	97 (70.3)	57 (59.4)	39 (40.6)		72 (74.2)	25 (25.8)		N/A	N/A	

### PrEP awareness

In multivariable analysis (Table 2), factors associated with an increased odds of PrEP awareness include residing in Abuja [adjusted odds ratio (aOR) 5.02; 95% confidence interval (CI): 2.13 to 11.83] and Lagos (aOR 12.30; 95% CI: 4.92 to 30.67) vs. residing in Plateau, living with HIV (aOR 2.56; 95% CI: 1.54 to 4.72), using location-based apps for seeking sexual partners (aOR 4.06; 95% CI: 2.28 to 7.24), having health insurance (aOR 2.31; 95% CI: 1.08 to 4.40), history of suicidal thoughts (aOR 2.05; 95% CI: 1.02 to 4.10), and history of PrEP use (aOR 45.5; 95% CI: 5.60 to 370.04).

**Table 2:** Unadjusted and adjusted associations with PrEP awareness, willingness to use PrEP, and history of PrEP use among Nigerian GBMSM from March-June 2019.

	Awareness of PrEP (n = 401)		Willingness to Use PrEP (n = 251)		PrEP Use (n = 138)	
	Unadjusted OR (95% CI)	Adjusted OR (95% CI)	Unadjusted OR (95% CI)	Adjusted OR (95% CI)	Unadjusted OR (95% CI)	Adjusted OR (95% CI)
**Site**						
Abuja	8.57 (4.41–16.64) ^§^	5.02 (2.13–11.83)*	0.64 (0.25–1.66)		1.26 (0.60–2.65)	
Delta	1.56 (0.80–3.03) ^§^	1.16 (0.52–2.62)	1.29 (0.48–3.47)		N/A	
Lagos	17.56 (8.56–36.01) ^§^	12.3 (4.92–30.67) ^§^	0.87 (0.35–2.14)		Ref	
Plateau	Ref	Ref	Ref		N/A	
***Socio-Demographics***						
Age Mean (SD)	1.01 (0.97–1.04)		0.95 (0.89–0.99)*	1.04 (0.99–1.10)	0.95 (0.89–1.02)	
**Relationship Status**						
Single	1.67 (1.11–2.51)*	1.13 (0.64–2.01)	1.69 (0.90–3.16)		1.61 (0.72–3.60)	
Not Single	Ref	Ref	Ref		Ref	
**Education**						
SSS or lower	Ref		Ref		Ref	
Some Uni or Vocation Sch	1.16 (0.69–1.94)		1.19 (0.50–2.82)		1.01(0.34–3.03)	
College degree or higher	4.26 (2.51–7.23)		0.88 (0.42–1.87)		1.18 (0.50–2.75)	
Other	1.87 (0.79–4.42)		0.47 (0.16–1.39)		2.24 (0.60–8.37)	
**Sexual Orientation**						
Gay/Homosexual	Ref		Ref		Ref	
Bisexual	1.26 (0.84–1.88)		0.72 (0.38–1.35)		1.25 (0.59–2.66)	
**Monthly Income (in Naira)**						
0–10000	Ref		Ref		Ref	
10,000–30,000	0.81 (0.47–1.40)		0.96 (0.36–2.57)		1.95 (0.65–5.82)	
30,000–50,000	1.33 (0.74–2.40)		0.54 (0.20–1.42)		2.46 (0.79–7.65)	
50,000–100,000	1.77 (0.91–3.44)		0.68 (0.21–2.19)		1.49 (0.39–5.67)	
100,000+	2.62 (1.28–5.34)*		0.36 (0.13–1.01)		0.85 (0.24–3.08)	
**Employment Status**						
Employed	0.76 (0.46–1.26)		0.83 (0.37–1.85)		1.14 (0.49–2.64)	
Unemployed	Ref		Ref		Ref	
**Financial Hardship**						
Yes	0.95 (0.63–1.42)		1.32 (0.70–2.47)		1.61 (0.75–3.45)	
No	Ref		Ref		Ref	
**History of Incarceration**						
Yes	0.93 (0.58–1.50)		1.87 (0.69–5.05)		0.28 (0.06–1.31)	
No	Ref		Ref		Ref	
***Sexual Health***						
**Sexual Position**						
Bottom/Versatile Bottom	1.16 (0.72–1.89)		1.46 (0.65–3.28)		0.75 (0.30–1.86)	
Versatile	1.10 (0.69–1.77)		1.28 (0.61–2.70)		1.60 (0.64–4.04)	
Versatile Top/Top	Ref		Ref		Ref	
**Self-Reported HIV Status**						
Positive/Unknown	1.54 (1.02–2.32)*	**2.56 (1.54–4.72)** ^§^	N/A		N/A	
Negative	Ref	Ref	N/A		N/A	
**Any History of STIs**						
Yes	1.41 (0.95–2.10)		1.02 (0.54–1.92)		1.64 (0.76–3.53)	
No	Ref		Ref		Ref	
**STIs in last year**						
Yes	1.12 (0.73–1.70)		0.92 (0.47–1.82)		2.09 (0.90–4.82)	
No	Ref		Ref		Ref	
**# of Receptive Anal sex acts in the last 30 days**						
0	Ref	Ref	Ref		Ref	
1	1.52 (0.83–2.79)	1.61 (0.67–3.84)	1.08 (0.43–2.76)		1.95 (0.61–6.20)	
2–3	1.11 (0.66–1.87)	0.93 (0.43–1.99)	1.38 (0.60–3.19)		2.29 (0.91–5.80)	
4–5	1.23 (0.66–2.29)	0.63 (0.27–1.50)	0.95 (0.35–2.59)		0.75 (0.19–2.97)	
6+	3.63 (1.49–8.84)*	1.90 (0.56–6.45)	1.22 (0.25–5.99)		1.95 (0.42–9.07)	
**# of Insertive Anal sex acts in the last 30 days**						
0	Ref		Ref		Ref	Ref
1	1.05 (0.55–2.01)		1.25 (0.45–3.47)		1.06 (0.28–4.06)	1.35 (0.32–5.66)
2–3	0.99 (0.59–1.67)		1.50 (0.64–3.49)		1.87 (0.66–5.33)	2.58 (0.80–8.30)
4–5	1.46 (0.77–2.75)		1.19 (0.45–3.15)		2.13 (0.61–7.39)	1.67 (0.46–6.08)
6+	1.34 (0.73–2.47)		0.90 (0.35–2.32)		5.19 (1.61–16.74)*	5.76 (1.58–20.98)*
**Condom Use last anal sex**						
Yes	**2.37 (1.45–3.87)**^§^	1.84 (0.90–3.76)	0.66 (0.28–1.59)		1.56 (0.48–5.06)	
No	Ref	Ref	Ref		Ref	
**Geosocial App Usage**						
Yes	**4.02 (2.65–6.09)** ^§^	**4.06 (2.28–7.24)** ^§^	1.05 (0.57–1.95)		0.96 (0.46–2.00)	
No	Ref	Ref	Ref		Ref	
***Healthcare Access***						
**Primary care Provider**						
Yes	1.32 (0.89–1.96)		1.16 (0.62–2.17)		1.16 (0.56–2.42)	
No	Ref		Ref		Ref	
**Health Insurance**						
Yes	**2.20 (1.23–3.93)** ^§^	**2.31 (1.08–4.40)** *	0.68 (0.32–1.47)		1.01 (0.40–2.55)	
No	Ref	Ref	Ref		Ref	
**Unable to assess medical care due to cost in last year**						
Yes	0.67 (0.45–0.99)*	1.17 (0.65–2.10)	0.79 (0.43–1.48)		0.65 (0.29–1.45)	
No	Ref	Ref	Ref		Ref	
***Mental Health***						
**Depressive Symptoms (CESD-20)**	1.01 (0.99–1.03)		1.04 (1.01–1.08)*	0.96 (0.92–0.99)*	1.01 (0.97–1.04)	
**Suicide Ideation**						
Yes	**2.24 (1.35–3.72)** ^§^	2.05 (1.02–4.10)*	1.54 (0.64–3.67)		0.61 (0.23–1.65)	
No	Ref	Ref	Ref		Ref	
**Internalized Homophobia**	**0.89 (0.84–0.95)** ^§^	0.96 (0.88–1.04)	0.99 (0.91–1.09)		1.00 (0.89–1.12)	
***Substance Use***						
**Hazardous Drinking (AUDIT-C)**						
Yes	1.62 (0.97–2.71)		0.62 (0.31–1.26)		1.12 (0.49–2.56)	
No	Ref		Ref		Ref	
**Lifetime Marijuana Use**						
Yes	1.40 (0.94–2.09)		0.63 (0.34–1.19)		1.05 (0.50–2.22)	
No	Ref		Ref		Ref	
**Lifetime Tramadol Use**						
Yes	1.31 (0.75–2.30)		0.97 (0.40–2.38)		2.31 (0.87–6.16)	
No	Ref		Ref		Ref	
**Lifetime Rohypnol Use**						
Yes	0.60 (0.34–1.04)		1.11 (0.46–2.69)		1.78 (0.67–4.74)	
No	Ref		Ref		Ref	
**Lifetime Codeine Use**						
Yes	0.72 (0.45–1.15)		0.73 (0.35–1.50)		1.94 (0.84–4.44)	
No	Ref		Ref		Ref	
**Lifetime Poppers Use**						
Yes	**4.51 (1.83–11.16)** ^§^	3.48 (0.83–14.62)	**0.30 (0.12–0.72)** ^§^	2.65 (0.84–8.36)	0.40 (0.11–1.45)	
No	Ref	Ref	Ref	Ref	Ref	
**Lifetime Cocaine Use**						
Yes	2.78 (1.15–6.70) ^§^	0.88 (0.22–3.56)	0.36 (0.14–0.93)*	1.77 (0.51–6.21)	1.61 (0.54–4.80)	
No	Ref	Ref	Ref	Ref	Ref	
***PrEP Indicators***						
**Awareness of PrEP**						
Aware of PrEP	N/A		0.65 (0.35–1.21)		**57.10 (7.80–417.97)** ^§^	**29.62 (3.78–231.84)** ^§^
Unaware of PrEP	N/A		Ref		Ref	Ref
**Willingness to use PrEP**						
Willing	0.65 (0.35–1.21)		N/A		1.97 (0.74–5.24)	
Not Willing/Not Sure	Ref		N/A		Ref	
**PrEP Use**						
History of PrEP Use	**26.68 (3.52–202.40)** ^§^	**45.53 (5.60–370.04)** ^§^	1.97 (0.74–5.24)		N/A	
No History of PrEP Use	Ref	Ref	Ref		N/A	

^§^ P<0.01

*P<0.05

### Willingness to use PrEP

In multivariable analysis (Table 2), decreasing clinically significant depressive symptoms was associated with lower willingness to use PrEP (aOR 0.96; 95% CI: 0.92 to 0.99).

### History of PrEP use

In the multivariable model (Table 2), factors associated with increased odds of having a history of PrEP use were those reporting 6 or more insertive anal sex acts in the last 30 days compared to those with none (aOR 5.76; 95% CI: 1.58 to 20.98) and being aware of PrEP (aOR 29.6; 95% CI: 3.78 to 231.84).

## Discussion

This is the first quantitative study that we are aware of to explore PrEP awareness, willingness to use PrEP, and history of PrEP use among GBMSM in Nigeria and West Africa. Nearly half (46.4%) of the Nigerian GBMSM in this study had no prior awareness of PrEP, but after being informed about its potential benefits, the majority (80.1%) were willing to use it. Furthermore, PrEP uptake among Nigerian GBMSM remains low (29.7%). Our findings align with a recent review article that found relatively low PrEP awareness (16.9–44.3%) but higher willingness to use PrEP (53.3–74.8%) among GBMSM in low and middle income countries [13].

We found that residing Lagos and Abuja was significantly associated with PrEP awareness compared to residing in Plateau. This finding is likely explained by the recent availability of PrEP among GBMSM in these two states.

Additionally, we found that people living with HIV had higher PrEP awareness compared to those who reported being HIV uninfected. Since recruitment occurred through CBOs, some of which provide HIV testing and counseling, and antiretroviral therapy, it is possible that people living with HIV may have been counselled on the efficacy of PrEP to decrease HIV transmission among serodiscordant sexual partnerships [14]. We found that individuals who had used location-based apps for seeking sexual partners had increased odds of being PrEP aware, compared to those who reported non-use. This finding support recommendation for using geosocial networking apps (e.g., Grindr, Badoo, etc) as potentially effective medium for dissemination of PrEP messaging as rollout expands in Nigeria, especially given thar more than half of the sample (53.4%) reported using these apps and these apps have been previously associated with greater number of sexual partners, condomless anal sex, and increased incidence of HIV and other sexually transmitted infections [15, 16]. This is especially relevant in Nigeria where homosexuality is criminalized and limited safe spaces exist for GBMSM, with many resorting to these apps to meet other GBMSM for a friendship, relationship, and/or sex.

GBMSM with clinically significant depressive symptoms had a decreased odds of willingness to use PrEP. This finding highlights how mental health issues might affect interest in PrEP uptake, which is particularly important as psychosocial health problems has been associated with increased risk for HIV infection among GBMSM [17–19]. A recently published study found that experiencing 4 or more psychosocial health problems (i.e. depressive symptoms, post-traumatic stress disorder, alcohol dependence, tobacco use, and hard-drug use)–compared to experience none or one psychosocial health problem–was significantly associated with increasing number of male sexual partners among Nigerian GBMSM [20]. Previous research has highlighted the need to screen for mental health problems among GBMSM PrEP users, as it may lead to suboptimal PrEP adherence [21] and overall health [22].

We found that having 6 or more insertive anal sex partners in the last 30 days and being aware of PrEP were significantly associated with history of PrEP use. This finding may provide evidence that the current ongoing demonstration trial of PrEP in Nigeria is capturing those who are at most risk and PrEP awareness translating to PrEP uptake. This finding highlights the need for a PrEP awareness campaign, on various platforms, that provides easily understood, evidence-based information about PrEP. This campaign will help increase health literacy both around PrEP specifically and more generally on various other topics related to sexual health Information about effectiveness, costs, and common side effects should be provided. The campaign should include testimonials trusted GBMSM community members, to add credibility and relatability of the health campaign. Also, key stakeholders such as GBMSM community opinion leaders, CBOs, and geosocial networking apps are major channels for dissemination of information about PrEP to Nigerian GBMSM. Intentional engagement of these groups, at every step, is pivotal to increasing PrEP knowledge among Nigerian GBMSM, which would facilitate PrEP uptake and adherence.

These findings should be examined in the context of some study limitations The cross-sectional design limits our ability to draw causal inferences from our findings. In addition, participants were mainly recruited through GBMSM community-based organizations and GBMSM social networks, thus findings may not be generalizable to GBMSM who do not seek services at these CBOS or are outside of these social networks. In addition, many of the measures relied on participant recall/self-report (e.g., HIV status); future studies should use more objective measurements when possible.

Despite these limitations, this is among the first known quantitative analyses of PrEP awareness/acceptability among Nigerian GBMSM. Additionally, this is the first study to sample GBMSM from four distinct regions of the country, which provided for a diverse sample of varied experiences.

## Conclusions

Taken together, we found that nearly half of the Nigerian GBMSM in this study had no prior awareness of PrEP, but after being informed about its potential benefits, the majority were willing to use it. Furthermore, PrEP uptake among Nigerian GBMSM remains low. Findings suggest that educational messages are necessary to ensure appropriate PrEP scale-up, especially tailored towards Nigerian GBMSM.

## References

[1] HIV/AIDS JUNPo, UNAIDS D. Geneva; 2017. 2017.

[2] Vu L, Adebajo S, Tun W, Sheehy M, Karlyn A, Njab J, et al High HIV prevalence among men who have sex with men in Nigeria: implications for combination prevention. JAIDS Journal of Acquired Immune Deficiency Syndromes. 2013;63(2):221–7. 10.1097/QAI.0b013e31828a3e60 23406978

[3] Smith DK, Herbst JH, Rose CE. Estimating HIV protective effects of method adherence with combinations of preexposure prophylaxis and condom use among African American men who have sex with men. Sexually transmitted diseases. 2015;42(2):88–92. 10.1097/OLQ.0000000000000238 . 25585067

[4] Donnell D, Baeten JM, Bumpus NN, Brantley J, Bangsberg DR, Haberer JE, et al HIV protective efficacy and correlates of tenofovir blood concentrations in a clinical trial of PrEP for HIV prevention. J Acquir Immune Defic Syndr. 2014;66(3):340–8. 10.1097/QAI.0000000000000172 24784763 PMC4059553

[5] Grant RM, Lama JR, Anderson PL, McMahan V, Liu AY, Vargas L, et al Preexposure chemoprophylaxis for HIV prevention in men who have sex with men. The New England journal of medicine. 2010;363(27):2587–99. 10.1056/NEJMoa1011205 21091279 PMC3079639

[6] Baral S, Scheibe A, Sullivan P, Trapence G, Lambert A, Bekker LG, et al Assessing Priorities for Combination HIV Prevention Research for Men Who have Sex with Men (MSM) in Africa. AIDS Behav. 2012 10.1007/s10461-012-0202-5 . 22610371

[7] AVAC. Nigeria PrEPWatch 2019 August 18, 2019.

[8] Ogunbajo A, Kang A, Shangani S, Wade RM, Onyango DP, Odero WW, et al Awareness and Acceptability of pre-exposure prophylaxis (PrEP) among gay, bisexual and other men who have sex with men (GBMSM) in Kenya. AIDS care. 2019:1–8. 10.1080/09540121.2019.1612023 PMC6663573 31039628

[9] Ogunbajo A, Leblanc NM, Kushwaha S, Boakye F, Hanson S, Smith MD, et al Knowledge and Acceptability of HIV pre-exposure prophylaxis (PrEP) among men who have sex with men (MSM) in Ghana. AIDS care. 2019:1–7. 10.1080/09540121.2019.1675858 PMC6980967 31597455

[10] Eaton WW, Smith C, Ybarra M, Muntaner C, Tien A. Center for Epidemiologic Studies Depression Scale: review and revision (CESD and CESD-R). 2004.

[11] Outland PL. Developing the LGBT minority stress measure: Colorado State University Libraries; 2016.

[12] Bush K, Kivlahan DR, McDonell MB, Fihn SD, Bradley KA. The AUDIT alcohol consumption questions (AUDIT-C): an effective brief screening test for problem drinking. Archives of internal medicine. 1998;158(16):1789–95. 10.1001/archinte.158.16.1789 9738608

[13] Yi S, Tuot S, Mwai GW, Ngin C, Chhim K, Pal K, et al Awareness and willingness to use HIV pre-exposure prophylaxis among men who have sex with men in low-and middle-income countries: a systematic review and meta-analysis. Journal of the international Aids Society. 2017;20(1):21580 10.7448/IAS.20.1.21580 28691439 PMC5515024

[14] Organization WH. Guidance on oral pre-exposure prophylaxis (PrEP) for serodiscordant couples, men and transgender women who have sex with men at high risk of HIV: recommendations for use in the context of demonstration projects, July 2012. 2012. 23586123

[15] Beymer MR, Weiss RE, Bolan RK, Rudy ET, Bourque LB, Rodriguez JP, et al Sex on demand: geosocial networking phone apps and risk of sexually transmitted infections among a cross-sectional sample of men who have sex with men in Los Angeles County. Sex Transm Infect. 2014;90(7):567–72. 10.1136/sextrans-2013-051494 24926041 PMC4198579

[16] Queiroz AAFLN, de Sousa ÁFL, de Araújo TME, de Oliveira FBM, Moura MEB, Reis RK. A review of risk behaviors for HIV infection by men who have sex with men through geosocial networking phone apps. Journal of the Association of Nurses in AIDS Care. 2017;28(5):807–18. 10.1016/j.jana.2017.03.009 28456472

[17] Safren SA, Reisner SL, Herrick A, Mimiaga MJ, Stall R. Mental health and HIV risk in men who have sex with men. Journal of acquired immune deficiency syndromes (1999). 2010;55(Suppl 2):S74. 21406991 10.1097/QAI.0b013e3181fbc939 PMC3074520

[18] Stall R, Mills TC, Williamson J, Hart T, Greenwood G, Paul J, et al Association of co-occurring psychosocial health problems and increased vulnerability to HIV/AIDS among urban men who have sex with men. American journal of public health. 2003;93(6):939–42. 10.2105/ajph.93.6.939 12773359 PMC1447874

[19] Mimiaga MJ, O’CLEIRIGH C, Biello KB, Robertson AM, Safren SA, Coates TJ, et al The effect of psychosocial syndemic production on 4-year HIV incidence and risk behavior in a large cohort of sexually active men who have sex with men. Journal of acquired immune deficiency syndromes (1999). 2015;68(3):329. 25501609 10.1097/QAI.0000000000000475 PMC4415161

[20] Ogunbajo A, Oke T, Jin H, Rashidi W, Iwuagwu S, Harper GW, et al A syndemic of psychosocial health problems is associated with increased HIV sexual risk among Nigerian gay, bisexual, and other men who have sex with men (GBMSM). AIDS care. 2019:1–6. 10.1080/09540121.2019.1678722 PMC6980916 31608657

[21] Mehrotra ML, Glidden DV, McMahan V, Amico KR, Hosek S, Defechereux P, et al The effect of depressive symptoms on adherence to daily oral PrEP in men who have sex with men and transgender women: a marginal structural model analysis of the iPrEx OLE study. AIDS and Behavior. 2016;20(7):1527–34. 10.1007/s10461-016-1415-9 27125241 PMC4912836

[22] Tan DH, Leon-Carlyle M, Mills R, Moses E, Carvalhal A. Self-administered screening for syndemic mental health problems should be routinely implemented among MSM PrEP users. Journal of Gay & Lesbian Mental Health. 2016;20(1):13–20.

